# Overexpressed somatic alleles are enriched in functional elements in Breast Cancer

**DOI:** 10.1038/s41598-017-08416-w

**Published:** 2017-08-15

**Authors:** Paula Restrepo, Mercedeh Movassagh, Nawaf Alomran, Christian Miller, Muzi Li, Chris Trenkov, Yulian Manchev, Sonali Bahl, Stephanie Warnken, Liam Spurr, Tatiyana Apanasovich, Keith Crandall, Nathan Edwards, Anelia Horvath

**Affiliations:** 1 Department of Pharmacology and Physiology, School of Medicine and Health Sciences, The George Washington University, Washington, DC 20037 USA; 2 McCormick Genomics and Proteomics Center, School of Medicine and Health Sciences, The George Washington University, Washington, DC 20037 USA; 3 University of Massachusetts Medical School, Program in Bioinformatics and Integrative Biology, Worcester, MA 01605 USA; 4 Department of Biochemistry and Molecular and Cellular Biology, Georgetown University, School of Medicine, Washington, DC 20057 USA; 5 Computational Biology Institute, The George Washington University, Washington, DC 20037 USA; 6 Department of Statistics, The George Washington University, Washington, DC 20037 USA; 7 Department of Biochemistry and Molecular Medicine, School of Medicine and Health Sciences, The George Washington University, Washington, DC 20037 USA

## Abstract

Asymmetric allele content in the transcriptome can be indicative of functional and selective features of the underlying genetic variants. Yet, imbalanced alleles, especially from diploid genome regions, are poorly explored in cancer. Here we systematically quantify and integrate the variant allele fraction from corresponding RNA and DNA sequence data from patients with breast cancer acquired through The Cancer Genome Atlas (TCGA). We test for correlation between allele prevalence and functionality in known cancer-implicated genes from the Cancer Gene Census (CGC). We document significant allele-preferential expression of functional variants in CGC genes and across the entire dataset. Notably, we find frequent allele-specific overexpression of variants in tumor-suppressor genes. We also report a list of over-expressed variants from non-CGC genes. Overall, our analysis presents an integrated set of features of somatic allele expression and points to the vast information content of the asymmetric alleles in the cancer transcriptome.

## Introduction

The cancer phenotype is largely driven by somatic mutations, whose carcinogenic effects are ultimately intervened by the transcription process^1–3^. As a mediator between genotype and phenotype, the tumor transcriptome reflects both advantage- selective pressure, and direct effects of the mutations on the transcription process. Hence, the tumor transcriptome is highly informative about the somatic functionality, especially through allele-specific approaches that can confine expressed structures to particular mutant alleles^1–4^.

Several studies have explored the allele-specific transcriptional landscape of cancer^1, 5–10^. Preferentially expressed alleles are reported to play a role in epithelial ovarian cancer^7^, as well as in microRNA-implicated carcinogenesis, an example of which is miR-31 dysregulation in lung cancer^8^. Imbalanced allele expression can be caused by both large chromosomal alterations, such as copy number alterations (CNAs), and single nucleotide somatic mutations^1^.

Nucleotide somatic mutations can affect the transcriptome through alteration of regulatory, splicing, or expression-rate modifying sites. Such effects commonly manifest in cis-fashion and directly impact the transcript abundance of the mutation bearing allele^1, 11, 12^. Mutations can also indirectly imbalance the allele content through changing the protein functions to either advance or impair the tumor growth. Functional mutations that provide selective advantage are referred to as drivers, and they are commonly targeted by either positive or negative selection forces to retain or deplete the growth-affecting allele^13–16^. Accordingly, somatic allele imbalance, including the extremes of loss or over-expression, can indicate tumorigenic functionality.

Expression imbalance of point mutations is particularly informative for regions with no CNAs, where potential effects on the transcription can be directly linked to the underlying nucleotide change^14^. Therefore, quantitative integration of allele signals between same-source DNA and RNA is instrumental for tracking chromosome-of-origin effects. The latter, in turn, can be used to search for new genes whose allele behavior follows the pattern of known cancer drivers and is thus indicative for potential carcinogenicity. Therefore, the few studies that quantitatively integrate allele abundance from matching DNA and RNA sequencing sources are very informative^10^.

Herein, we apply a software that we recently developed – RNA2DNAlign^9^ – to systematically quantify the allele expression of somatic variants in breast cancer samples from The Cancer Genome Atlas (TCGA). RNA2DNAlign counts variant and reference sequencing reads derived from compatible RNA and DNA datasets, and tests for allelic imbalance; it also calls positions with extreme allele distributions, including somatic over-Expression (SOM-E) or loss (SOM-L). We compute and compare the somatic variant allele fraction (VAF) of mutations in genes from the Cancer Gene Census (CGC)^17^ to those in the rest of the genes in our samples. We also report a list of non-CGC genes with over-expressed somatic variants. Overall, we present an integrated set of somatic allele-specific expression features, in the context of their potential underlying functionality.

## Results

### Strategy

**Figure 1:**
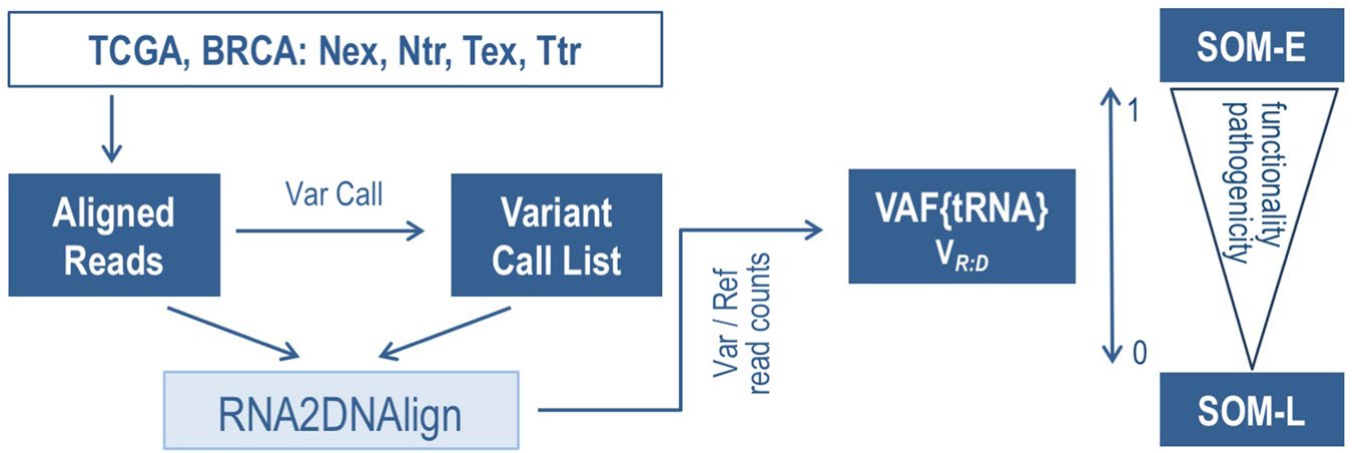
Major steps of the analysis of allele distribution for somatic variants in our dataset. V_R:D_ was analyzed for correlation with different functional mutations groups in oncogenes, tumor suppressors, and the rest of the genes. SOM-E and SOM-L variants were compared with the rest of the somatic mutations for predicted pathogenicity and location in functional motifs such as transcription and splice factor binding sites, and highly preserved sequences.

**Figure 2:**
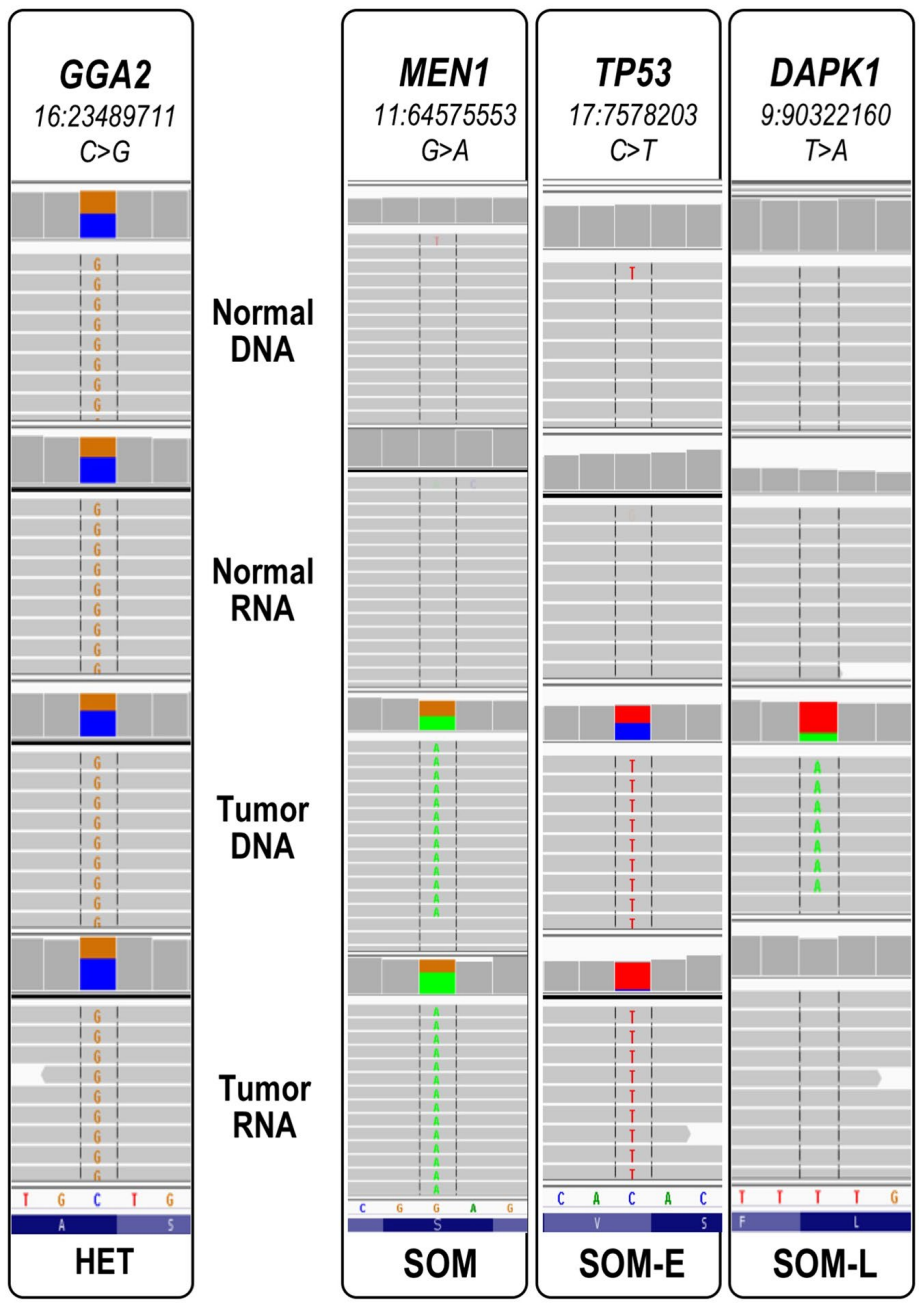
IGV visualization of somatic mutations that are over-expressed (SOM-E, middle) or under-expressed (SOM-L, right) compared to expected allele distribution for a germline heterozygote variant (left); the heterozygosity is reflected through color-coding of the summary flag on the top of each panel. The gray lines represent reads, and the colored letters show differences from the reference.

Our strategy was to first systematically quantify the variant allele fraction of the tumor RNA (VAF{tRNA}), and then to assess for correlation between RNA allele asymmetry and functional features (Fig. 1). Somatic variants (SOM) with a bi-allelic signal in the tumor DNA and a mono-allelic signal in the tumor RNA were classified as SOM-L (VAF{tRNA} ~ 0) or SOM-E (VAF{tRNA} ~ 1; Fig. 2). We assess both absolute VAF{tRNA}, and relative to VAF{tDNA}, for which we introduce the expression V_R:D_ = VAF{tRNA}:VAF{tDNA}. We note that through accounting for the VAF{tDNA}, V_R:D_ reflects the overall genome composition of the sample, including the contribution from large rearrangements, and admixture with non-tumor genomes (i.e. the sample purity). First, we analyzed the allele distribution for mutations in known oncogenes and tumor suppressors from CGC. We evaluated VAF{tRNA} and V_R:D_ for correlation with functional features including conservation, predicted pathogenicity, and location in critical sequence motifs. Next, we assessed these features, in the context of their allelic expression, in the non-CGC dataset, and highlighted variants whose somatic allele patterns follow functionality-associated allele behavior of known cancer drivers.

### Overall dataset characteristics

**Figure 3:**
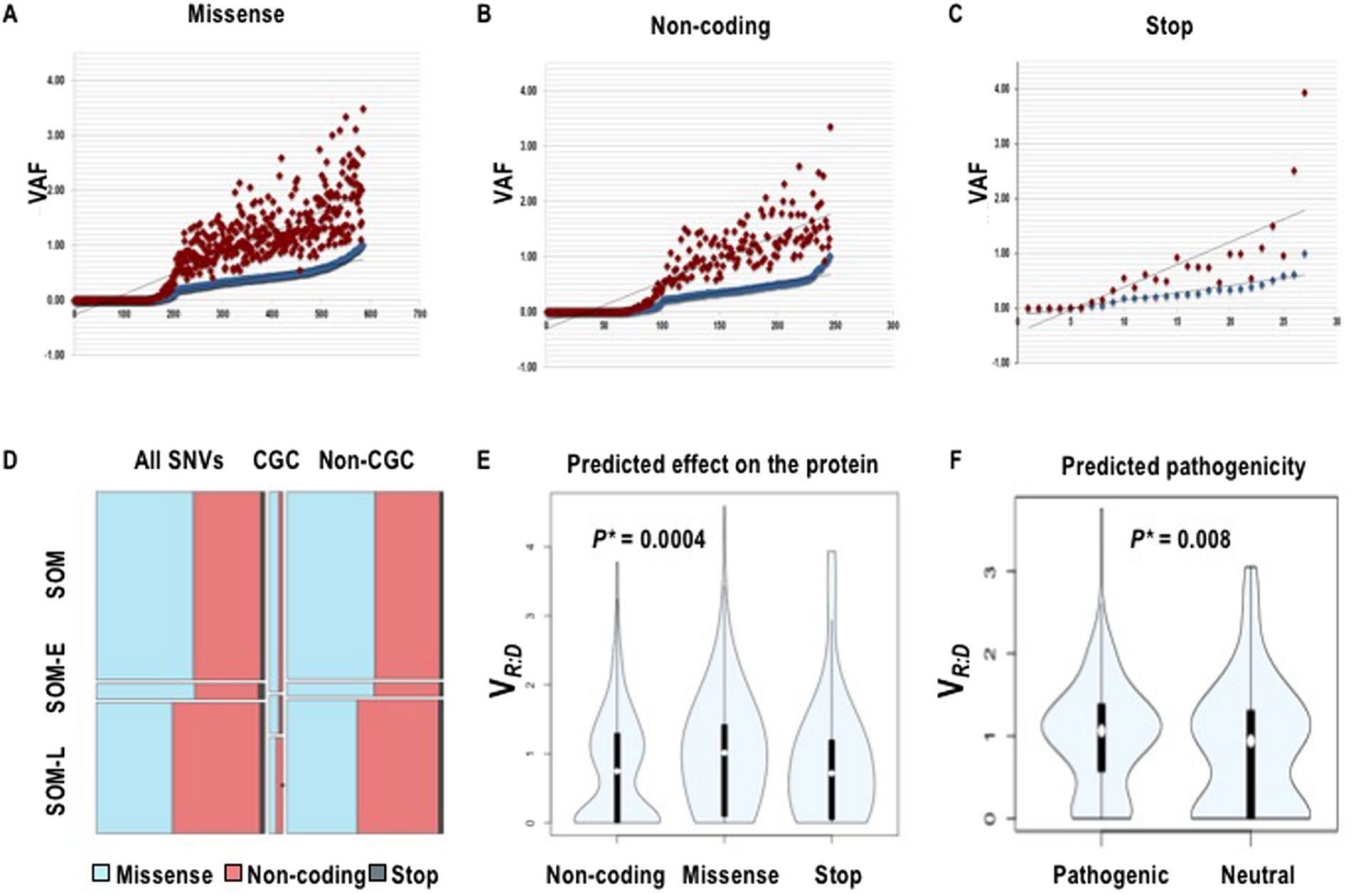
**(A–C)** Distribution of VAFtRNA (blue) and V_R:D_ (red) in the subgroups of missense (**A**), non-coding (**B**) and stop-codon variants. The X axis shows the number of variants in each functional category. Positive correlation is seen in all three mutation groups. (**D**) Distribution of SOM-E and SOM-L expression status in regards to predicted effect on the protein function in the entire set, CGC-, and non-CGC variants. (**E**) V_R:D_ for non-coding, missense and stop-codon variants across the entire dataset. Clearly different V_R:D_ distribution is seen among the different functional subtypes, with the missense mutations showing higher V_R:D_, indicative for higher allele expression of potentially functional transcripts. (**F**) V_R:D_ for pathogenic and neutral variants as predicted by FATHMM. The difference in the distribution is due to the larger proportion of the pathogenic mutations with higher V_R:D_.

A total of 1238 (1139 unique) mutations in 921 genes, from which 68 were listed in CGC, satisfied the requirements for our analysis (Supplementary Table 1 and Supplementary Figure 1). Between 7 and 51 somatic point mutations in expressed coding regions were assessed per individual sample. Most of the mutations (94%) were singletons (present in only one sample), whereas 44 mutations were seen in 2, 12 in 3, 4 in 4, 2 in 5, and one mutation each was found in 6 and 7 different samples. Notably, all non-singleton mutations shared similar allele expression status across the different samples. A total of 437 somatic mutations (38.3%) were not expressed at all in the transcriptome (SOM-L), and 73 mutations (4.9%) were over-expressed (SOM-E). The analysis of the variant allele fraction showed an overall positive correlation between VAF{tDNA} and VAF{tRNA} (Spearman correlation r = 0.38, Fig. 3A–C). The functional distribution of the predicted consequences on the protein, and the intersection with their allele-expression status is presented on Fig. 3D. The missense, non-coding and stop-codon variants showed clearly different patterns of V_R:D_ with a higher V_R:D_ in the missense mutations, as compared to the non-coding and stop-codon variants (p = 0.0004, Kruskal-Wallis test^18^, Fig. 3E). Notably, we observed distribution towards higher V_R:D_ of the variants predicted to be pathogenic through FATHMM (Functional Analysis Through Hidden Markov Models), Fig. 3F ^19, 20^.

### CGC genes somatic allele expression: overall features

The 68 known cancer driver genes collectively contained 103 (88 unique) somatic mutations qualifying for the analysis (Supplementary Table 2)^17^. Mutations in *PIK3CA*, *MITF*, *ACVR2A*, *CLIP1*, and *TCEA1* were called in more than one sample. In this gene-set, we called 10 SOM-E variants: seven missense substitutions, two synonymous variants, and, notably, the stop-codon R63X in *CDH1*. Of note, four of the SOM-E missense substitutions were called in *TP53* (See Supplementary Table 2). A higher number - 25 - SOM-L variants were completely absent from the transcriptome in the CGC dataset.

**Figure 4:**
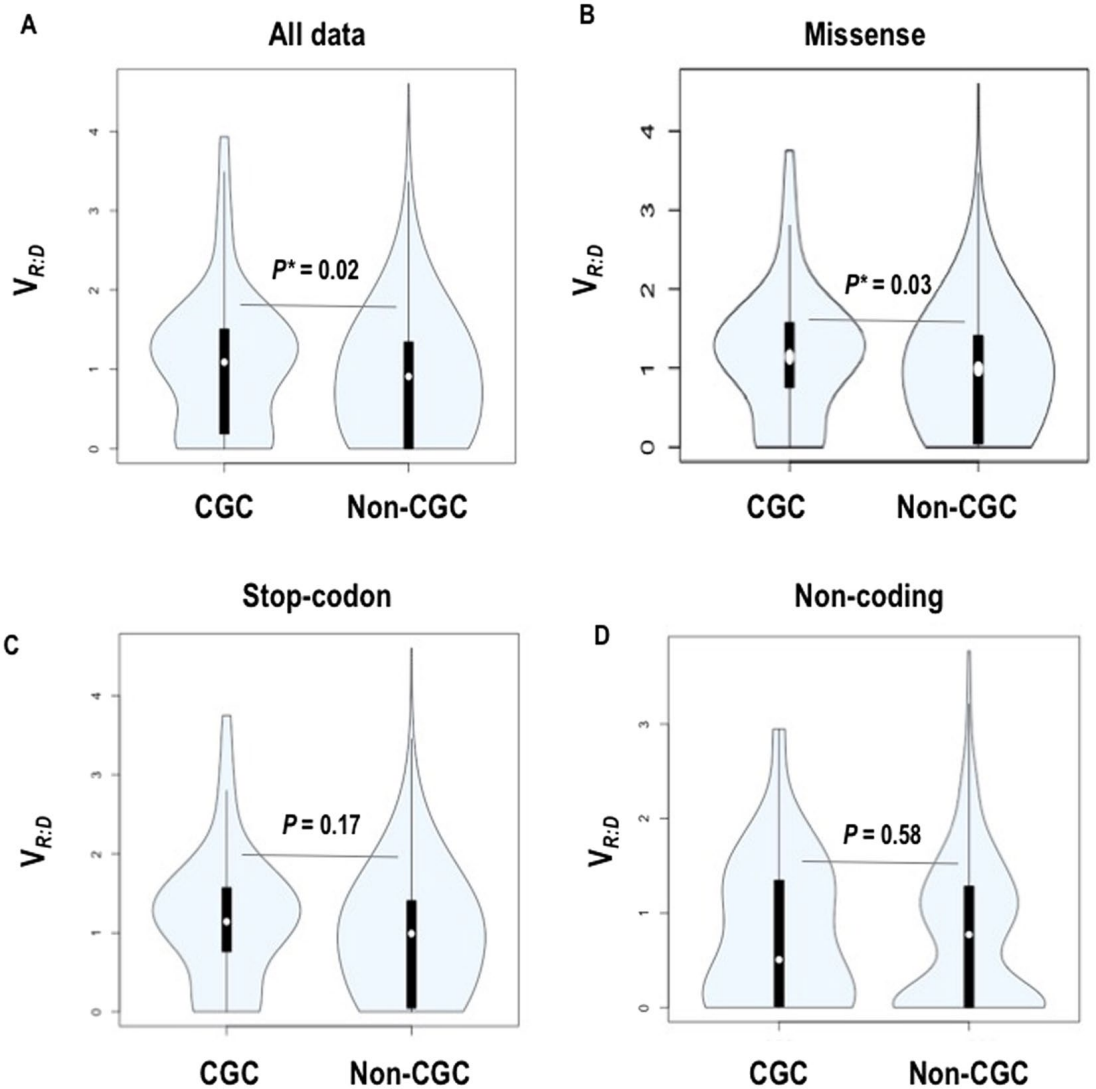
V_R:D_ in the CGC vs non-CGC genes (**A**), in missense variants (**B**), in stop-codon variants (**B**), and in non-coding variants.

Several noticeable observations were made in the CGC subset. First, different V_R:D_ distribution was observed in the CGC variants as compared to the rest of the dataset (p = 0.02, Kruskal-Wallis test^18^, Fig. 4A); the difference due to larger proportion of CGC variants with higher allele expression. Second, the CGC missense mutations showed higher allele expression as compared to the missense mutations in the entire dataset (p = 0.03, Kruskal-Wallis test^18^, Fig. 4B). Notably, a tendency for higher V_R:D_ was also seen for the stop-codon mutations, albeit not reaching statistical significance (Fig. 4C). In contrast, the non-coding variants did not show significant differences between the CGC and non-CGC genes (Fig. 4D). Third, we documented positive correlation between V_R:D_ and predicted pathogenicity assessed by the CADD score (Combined Annotation Dependent Depletion)^21^, (Spearman r = 0.25), FATHMM score (Functional Analysis Through Hidden Markov Models)^19, 20^ (Spearman r = 0.17), and conservation of the position of the somatic mutation as assessed through GERP (Genomic Evolutionary Rate Profiling, Spearman r = 0.29)^22–26^. Of note, 21% of the variants in the CGC dataset modeled through FATHMM as pathogenic have been reported in cancer-based studies^17^. Collectively, all the above analyses supported preferential expression of functional alleles in the CGC dataset.

We then assessed CGC SOM-E and SOM-L mutations in the context of their harboring gene’s function and mechanism of action. The first noticeable observation was a tendency for over-representation of genes acting in recessive molecular mode among the SOM-E variants, as opposed to more-frequent dominant mode of action in the genes bearing SOM-L variants (p = 0.15). Recessive mode is traditionally more often associated with tumor-suppressive function, while dominant action is reported frequently for oncogenes^27^. In our study, SOM-E status appears not to result from a genomic DNA loss, as evident by the tumor DNA’s biallelic signal (0 < VAF{tDNA} < 1). Both the inhibition of the reference and the enhancement of the mutant allele’s expression could result in mutant RNA dominance, and these effects could be independent or related to the functionality of the particular mutation. In the case of the mutations acknowledged as pathogenic in suppressor genes, the observed overexpression is consistent with mutation-driven allele inactivation, possibly favored by positive selection forces. Such interpretation is in line also with the over-expressed stop-codon R63X in *CDH1* ^28^.

For the SOM-L mutations, whether their expressional loss is linked to potential oncogenic action of the host gene, is to be determined on per-gene basis. It is important to recognize that many somatic variants are randomly lost in the tumor transcriptome, and the number of transcribed ones can depend on factors such as Estrogen Receptor (ER) expression levels^1^. While it is possible for a SOM-L variant to reside on a lost allele by coincidence, this is unlikely to explain all SOM-L patterns for variants with known pathogenicity.

### Allele expression of somatic mutations in the non-CGC genes

The integrated features of somatic allele expression in the non-CGC genes is presented in Supplementary Table 3. We documented concurrent to the CGC dataset positive correlation between increased allele expression and predicted pathogenicity and conservation scores (Spearman CADD r = 0.11, FATHMM r = 0.12, and GERP r = 0.17 (Supplementary Table 3).

The non-CGC somatic mutations with strong overexpression of the mutation-bearing allele (VAF{tRNA} = 1) are presented in Table 1. We next assessed the SOM-E variants for location within transcription and splicing factor binding sites, including analysis for generation of a new binding site outside of known protein - recognizable sequences^29^. Indeed, 18 out of the 42 non-CGC SOM-E variants positioned outside an existing TFBS were predicted to generate a new motif recognizable by either transcription or a splicing factor^29, 30^.

**Table 1:** SOM-E mutations in non-GCG genes: location within transcription and splicing factor recognizable motifs.

Gene	Chr:pos (hg38)	Function	TFBS	SFBS
TMEM51	chr1:15215414C > A	missense	none	none
NBPF3	chr1:21481730T > C	non-coding	none	none to SRp40
EPHA10	chr1:37720517C > T	missense	none	none
KIF26B	chr1:245609349C > G	missense	none to V$LRH1_Q5_01	none
ILDR1	chr3:122001432G > A	non-coding	V$PPARG_02	none to Sam68, SLM-2
MUC20	chr3:195725818C > T	non-coding	V$CREB1_Q6	hnRNP DL, SRp55tonone
ZNF518B	chr4:10445288C > G	missense	V$PBX1_02	none
BBS7	chr4:121828063C > G	non-coding	none	hnRNP, HuB, MBNL1toTIA-1
OTUD4	chr4:145146395G > A	missense	none	SRP4 0to hnRNPA1
SH3RF1	chr4:169136534G > A	non-coding	none	MBNL1 to SRp40
SORBS2	chr4:185589715C > T	missense	none	YB-1 to SAM68
MYO10	chr5:16877688C > G	missense	V$YY1_01	none
MSH3	chr5:80768937T > A	missense	V$STAT3_01	none
PCDHB5	chr5:141136316C > T	non-coding	none	none
GRPEL2	chr5:149351223G > A	missense	V$YY1_02	none
TCOF1	chr5:150376236C > T	missense	none	SRp20/Nova-1/Nova-2 to none
MDN1	chr6:89700782A > T	non-coding	V$SMAD4_Q6_01	none
TNRC18	chr7:5316065C > A	non-coding	none	none
WDR60	chr7:158871385A > G	missense	none	none to SC35,SF2/ASF,hnRNPA1
FZD3	chr8:28527405G > A	non-coding	none	none
DAPK1	chr9:87706999C > T	missense	V$NFAT_Q6	none
COL27A1	chr9:114309301C > G	missense	none to V$MYOGENIN_Q6_01	none
PLCE1	chr10:94270600A > C	missense	none to V$NFAT1_Q4	SF2/ASF,hnRNPA1 to none
PDCD11	chr10:103441838A > C	missense	none	YB-1 to SRp-40
MUC6	chr11:1016406G > A	missense	none to V$NFAT1_Q4	none
ACER3	chr11:76861031G > T	missense	none	SRp30c to none
RAB38	chr11:88175236A > T	missense	V$PPARG_02	none
PHLDB1	chr11:118627958C > T	missense	V$IK3_01	none to HuB,TIA-1,SRp40
WNK1	chr12:753666C > G	missense	V$GFI1_01	none
NFE2	chr12:54292991G > A	missense	none to V$BEN_01	none to YB-1,SRp40
NUAK1	chr12:106067839A > T	missense	V$OCT1_06	none
RASAL1	chr12:113114816C > G	missense	V$YY1_01	none
SLITRK6	chr13:85795773C > A	missense	V$SMAD4_Q6_01	SF2/ASF,SRp38,YB-1 to Sam68
ATP11A	chr13:112858175C > A	missense	V$PAX5_01	none
NYNRIN	chr14:24411385C > G	non-coding	none to V$BEN_01	MBNL1
CLMN	chr14:95203587C > T	missense	none	none to hnRNPI
AHNAK2	chr14:104948892T > C	missense	none	none
RAD51	chr15:40706209C > A	non-coding	V$CEBPB_02	none
CCNB2	chr15:59125011G > A	non-coding	none	none
SULT1A2	chr16:28592021A > G	non-coding	none	SRp30c to none
NFATC3	chr16:68190983G > A	missense	none to V$GATA_Q6	none to SLM-2, Sam68
MED31	chr17:6651601A > G	non-coding	none	SRp30c to none
CHRNB1	chr17:7447082C > T	non-coding	none	none to ETR-3
ACBD4	chr17:45136583C > T	missense	none	SRp55t to SC35
ABCA7	chr19:1041510G > A	missense	none	none to YB-1, SRp20
LMNB2	chr19:2431813G > A	non-coding	none	SRp55 to SC35
ZNF676	chr19:22180184G > T	non-coding	none to V$NFAT1_Q4	deleted MBNL1
ZIM2	chr19:56774836G > T	stop	none to V$DRI1_01	none to Sam68, SLM-2
MRPL30	chr2:99181122C > A	non-coding	none to V$NFAT1_Q4	SLM-2 to hnRNP,DAZAP1, HuD
PASK	chr2:241126376C > G	missense	none	ETR-3 to SF2/ASF
TOP3B	chr22:21964200A > T	non-coding	none	hnRNPH1,hnRNPH2 to none
GGA1	chr22:37620258G > A	synonymous	none	ETR-3, SRp30c to hnRNPH1/2
RIBC2	chr22:45426055G > A	non-coding	none	hnRNP K to SF2/ASF
GRPR	chrX:16123978C > G	missense	none	none
TBC1D25	chrX:48560553C > G	missense	none	none
IGBP1	chrX:70133976C > T	missense	none	MNBL1 to SRp40, SRp55
HTATSF1	chrX:136510164G > C	missense	none	none to SRp20, YB-1

Next, we reviewed, on a per-gene basis, the current knowledge on the SOM-E genes and their possible implications in cancer. Despite not being listed in the CGC, some of these genes – such as *MSH3* and *NUAK1* and *NFE2* – have been repeatedly linked to cancer^31–33^. Notably, more of the SOM-E genes linked to tumor suppressor features (as opposed to oncogenic, p = 3.8e-4, Metacore), which we concurrently observed in the CGC dataset^34, 35^. Another striking observation is that 6 of the genes with SOM-E variants –*MSH3*, *RAD51*, *TCOF1*, *TP53BP1*, *CCNB2*, and *TOP3B* – are directly implicated in DNA damage response and repair^36–39^ which was also the top-enriched pathway in the SOM-E dataset (p = 0.05, Metacore). In contrast, the most represented pathway in the SOM-L group was the immune response (p = 0.05, Metacore). In regards to GO annotations, two differences were detected between the SOM-E and SOM-L groups (Supplementary Figure 2). First, SOM-E variants were more frequently located in genes encoding receptors and signal transducers, while a higher proportion of the SOM-L variants resided in structure-supportive genes. In regards to biological processes, the SOM-E group was enriched in genes involved in response to stimuli.

## Discussion

Ultimately, the accurate assessment of the expressed allele fraction is only possible in the context of the corresponding DNA alleles’ content. Herein, we integrate matching RNA and DNA allele fraction from bi-allelic DNA regions to identify transcriptome-favored alleles. We focus more specifically on somatic point mutations in breast cancer, which we assess for tumorigenic functionality that can underlie selective transcriptome preference.

The first striking observation from our study is that transcriptome-preferred alleles are enriched in functional features, which are often predicted to alter the original protein function. This correlation was stronger in the group of genes traditionally acknowledged as tumor suppressors. Tumor suppressors are often lost during progression, and their loss is considered a contribution to tumor growth^40^. In our data we see a strong expression preference towards somatically mutated tumor suppressor transcripts, including such bearing a premature stop-codon. Increased allele expression can be either directly caused by mutation-promoted cis transcription activation, or/and retention of the mutant allele in the transcriptome via positive selection. Both scenarios infer functionality and growth-supportive potential. Conforming with that, highly expressed somatic variants, including SOM-E, were more frequently located in highly conserved and predicted to be functional genomic sequences. Taken together, these data are consistent with gain-of-function mechanism favored by the tumor transcriptome. An active role of over-expressed variants is also supported by the selection for maintaining the expression of a complete, translation-ready transcripts, suggesting a possible role of the altered/shortened proteins in the tumor progression. Indeed, once recognized as tumor suppressors, many of the genes in our SOM-E set, including *TP53* are now acknowledged to play more complex roles that include oncogenic action^41–44^. Both inactivation and altering the protein function can be crucial for the tumor development. Regardless the mechanism of action, the above observations mark allelic overexpression as a highly informative metric that can be used to outline functionally enriched somatic datasets.

The proportion of SOM-L alleles in our data is generally consistent with other reports^1^. Under-expressed alleles, including SOM-L, also correlated with functional annotations and regulatory motifs, though did not reach the significance of SOM-E. In contrast to SOM-E, SOM-L variants confer features that imply intolerance of the transcriptional machinery to the harbored variant. In the absence of CNAs, several mechanisms could potentially lower allele expression levels of mutation bearing transcripts. A well acknowledged scenario is the surveillance-driven targeting of transcripts with deleterious variants, the most prominent example of which is NMD^1, 45^. A degradation mechanism can also take place where the mutation results in an unstable RNA structure^46^. Finally, a mutation can destroy a binding site for a transcription or splicing factor, thus directly abolishing the expression of the underlying alleles^14^. Additional factors, such as high ER expression levels, are also reported to correlate with a decreased number of expressed somatic mutations^1^. Besides the above mutation-focused mechanisms, SOM-L may result from random under-expression in the tumor transcriptome, and the general infidelity of cancer transcriptional machinery^47, 48^. The later confers higher contribution of randomness towards SOM-L loci, which is likely to dilute functional annotations in this group.

Another striking observation from our analysis is the expression pattern of stop-codon mutations. Several recent studies have published decreased expression of stop-codon bearing variants in cancer, and have linked it to NMD^1, 49^. Notably, in our data we see stop-codon bearing alleles over-represented as compared to the reference. Whether these expressed RNAs are translated into shorter proteins is subject of further studies, but this possibility is consistent by the presence of premature stop containing, translation-ready transcripts^1^. While NMD is knowledgeably impaired in cancer, our data suggests gene-selective NMD actions^50–52^.

Distinguishing pathogenic mutations from the more prevalent neutral variants constitutes one of the greatest challenges of cancer biology, leading to substantial effort towards developing confident analytic strategies. Modern methods integrate traditional frequency based approaches with expression abundance, functional effects, interaction networks and pathway context^13, 53–60^. Here, we integrate somatic allele fraction with most of the above strategies and the knowledge on tumor driving mechanisms, and evaluate the potential of asymmetric allele expression to predict cancer implicated variants. We document distinct allele signatures of cancer drivers at several levels. First, mutations in known cancer genes from our dataset presented more frequently with extreme allele patterns. An example is *TP53*, mutation in which were frequently either over-expressed or lost. Second, mutations in known cancer-implicated genes presented with higher allele expression. This was also reflected in the higher percentage of SOM-E variants among the known cancer genes. Third, SOM-E mutation sites were enriched in conservation and functional motifs. Cumulatively, these findings highlight the SOM-E status as a potential indicator for cancer-driving functionality. Based on the above, we list the non-CGC genes whose expression status follows the drivers-enriched SOM-E status (see Table 1); albeit not included in the CGC list, some of these genes have been linked to cancer before and are worth further investigation. In summary, our research illustrates an important correlation between asymmetric alleles and cancer-implicated functionality, and functionality in general, and underscores the vast information content of our strategy to systematically outline asymmetrically expressed alleles. This strategy is applicable to all types of cancer and is now enabled by the growing accessibility of matched DNA and RNA sequence data new tools for their integration and analysis^9, 61, 62^.

## Methods

### TCGA samples selection

We first identified all breast cancer samples for which the following five sets were available: normal exome, normal transcriptome, tumor exome, tumor transcriptome, and CNA data (segmentation file based on Affymetrix SNPv6 array profiling)^12, 60, 63, 64^. All these samples had at purity assessed with at least three of the following five purity estimators: ESTIMATE, ABSOLUTE, LUMP, IHC and the Consensus Purity Estimation (CPE)^65–68^. From these, we excluded samples with extensive (more than 3 standard deviations) number of somatic mutations, possibly due to clustered genomic rearrangements^69, 70^. The remaining 72 samples (Supplementary Table 4) were retained for further analysis. We reviewed the pathology reports and retrieved the available clinical information; data for 41 (57%) of the studied samples was available (See Supplementary Table 4). The highest proportion of the samples were ductal adenocarcinomas, either ER, or ER/PR positive. We did not observe any significantly distinguishing somatic expression patterns, which is likely due to the small sample size. The purity, as assessed by the above-mentioned algorithms, is shown in Supplementary Table 5.

### Allele count computation

All the used datasets were generated through paired-end sequencing on an Illumina HiSeq platform. The aligned to the human genome reference (hg38) sequencing reads (Binary Alignment Maps,bams) were downloaded from the Genomic Data Commons Data Portal (https://portal.gdc.cancer.gov/) and processed downstream through an in-house pipeline. Briefly, for both DNA and RNA datasets variants were called using the mpileup module of SAMtools^70^. The variants were further annotated through SeattleSeq. 147 (http://snp.gs.washington.edu/SeattleSeqAnnotation147/). The alignments together with the variant calls (.vcf) were processed through RNA2DNAlign. RNA2DNAlign produced variant and reference sequencing reads counts for all the variant positions in all four datasets (normal exome, normal transcriptome, tumor exome and tumor transcriptome). The read count assessments were visually examined using Integrative Genome Viewer^72^. We excluded from further analyses variants which (1) were covered with less than 10 sequencing read in the tumor DNA or the RNA sequencing data; (2) reside in known imprinted regions, and (3) reside in area affected by copy number change in the corresponding sample, as defined based on the CNA segmentation file, (4) were present in the normal DNA or RNA, suggestive for germline origin.

### Assessment for allele distribution

Allele expression rates within a sample were determined through estimation of the relative abundance of variant over total sequence read counts, expressed as Variant Allele Fraction (VAF). For each somatic mutation, we computed the VAF = n(var)/(n(ref) + n(var)), for both tumor RNA (VAF{tRNA}) and tumor DNA (VAF{tDNA}), where n(ref) and n(var) are the counts of the variant and reference sequencing reads covering the position. To account for allele asymmetries related to DNA, we analyzed VAF{tRNA} in the context of the corresponding VAF{tDNA}. Over-expression of somatic mutations (SOM-E status) was determined as prevalence of variant sequencing reads in the transcriptome (VAF{tRNA} ~ 1), while SOM-L was defined by complete loss of the mutant allele in the transcriptome (VAF{tRNA} ~ 0). All the VAF{tRNA} values were used in a correlation analyses to search for association with functional features. Overall VAFs across the studied datasets were illustrated using Circos plots (See Supplementary Figure 1)^73^.

### Functional and enrichment analyses

Functional annotations, conservation scores and modeled pathogenicity were extracted using the SeattleSeq annotation 147 (http://snp.gs.washington.edu/SeattleSeqAnnotation147/index.jsp). Pathogenicity was modeled using PolyPhen, CADD and FATHMM models, and Conservation was assessed based on Phast, GREP and Grantham Scores^20–26^. Gene Ontology categories, pathway enrichment and network analysis were assessed using Metacore (Claritive Analytics). Transcription factor binding cites were analyzed using TRANSFAC 7.0^29^ and splicing motifs were assessed using SpliceAid2^30^.

### Statistics

SOM, SOM-E and SOM-L variants were called based on a binomial test for variant and reference sequencing read distribution, as previously described^9^. The distributions of SOM-E and SOM-L across tumor-suppressors, oncogenes, and the rest of the genes in the datasets, as well as the distribution of functional elements across SOM, SOM-E and SOM-L, were assessed using the Fisher exact test, Pearson chi-square test, Kruskal-Wallis rank sum test, linear regression analysis, and the Spearman rank correlation coefficient^18, 74, 75^. Yates’s correction for continuity was applied for tests with less than 5 measurements in any category^76^. The means of the VAF across different mutation types were compared using Student’s t-test^77^. P-values below 0.05 were considered significant. For multiple trials, the significance value was corrected using Benjamini-Hochberg False Discovery Rate (FDR) technique.

## Electronic supplementary material

Supplementary Figures

Supplementary Tables 1–5

## References

[1] Shlien A (2016). Direct Transcriptional Consequences of Somatic Mutation in Breast Cancer. Cell Reports.

[2] Eswaran J (2013). RNA sequencing of cancer reveals novel splicing alterations. Scientific reports.

[3] Horvath A (2013). Novel insights into breast cancer genetic variance through RNA sequencing. Scientific reports.

[4] Fredriksson NJ, Ny L, Nilsson JA, Larsson E (2014). Systematic analysis of noncoding somatic mutations and gene expression alterations across 14 tumor types. Nature Genetics.

[5] Lin W (2017). Allelic expression imbalance polymorphisms in susceptibility chromosome regions and the risk and survival of breast cancer. Molecular Carcinogenesis.

[6] French, J. & Edwards, S. Allelic imbalance in human breast cancer. *Oncotarget* **8** (2017). 10.18632/oncotarget.14648 PMC5355213 28099932

[7] Halabi NM (2016). Preferential Allele Expression Analysis Identifies Shared Germline and Somatic Driver Genes in Advanced Ovarian Cancer. PLoS Genetics.

[8] Okudela K (2014). Allelic imbalance in the miR-31 host gene locus in lung cancer–its potential role in carcinogenesis. Plos one.

[9] Movassagh, M. *et al*. RNA2DNAlign: nucleotide resolution allele asymmetries through quantitative assessment of RNA and DNA paired sequencing data. *Nucleic Acids Research* (2016). 10.1093/nar/gkw757 PMC5159535 27576531

[10] Rhee, J.-K., Lee, S., Park, W.-Y., Kim, Y.-H. and Kim, T.-M. Allelic imbalance of somatic mutations in cancer genomes and transcriptomes. *Sci. Rep.* **7** (2017). 10.1038/s41598-017-01966-z PMC5431982 28490743

[11] Wittkopp PJ, Kalay G (2011). Cis-regulatory elements: molecular mechanisms and evolutionary processes underlying divergence. Nature reviews. Genetics.

[12] Ding J (2015). Systematic analysis of somatic mutations impacting gene expression in 12 tumour types. Nature communica- tions.

[13] Van den Eynden J, Fierro AC, Verbeke LPC, Marchal K (2015). SomInaClust: detection of cancer genes based on somatic mutation patterns of inactivation and clustering. BMC bioinformatics.

[14] Vorontsov IE (2016). Negative selection maintains transcription factor binding motifs in human cancer. BMC genomics.

[15] Kern SE, Winter JM (2006). Elegance, silence and nonsense in the mutations literature for solid tumors. Cancer biology & therapy.

[16] Castro-Giner F, Ratcliffe P, Tomlinson I (2015). The mini-driver model of polygenic cancer evolution. Nature reviews. Cancer.

[17] Forbes SA (2015). COSMIC: exploring the world’s knowledge of somatic mutations in human cancer. Nucleic acids research.

[18] Kruskal WH, Wallis WA (1952). Use of ranks in one-criterion variance analysis. Journal of the American Statistical Association.

[19] Shihab HA (2013). Predicting the functional, molecular, and phenotypic consequences of amino acid substitutions using hidden Markov models. Human mutation.

[20] Shihab HA (2014). Ranking non-synonymous single nucleotide polymorphisms based on disease concepts. Human genomics.

[21] Kircher M (2014). A general framework for estimating the relative pathogenicity of human genetic variants. Nature genetics.

[22] Grantham R (1974). Amino acid difference formula to help explain protein evolution. Science (New York, N.Y.).

[23] Cooper GM (2005). Distribution and intensity of constraint in mammalian genomic sequence. Genome research.

[24] Hubisz MJ, Pollard KS, Siepel A (2011). PHAST and RPHAST: phylogenetic analysis with space/time models. Briefings in bioinformatics.

[25] Siepel A (2005). Evolutionarily conserved elements in vertebrate, insect, worm, and yeast genomes. Genome research.

[26] Siepel A, Haussler D (2004). Phylogenetic estimation of context-dependent substitution rates by maximum likelihood. Molecular biology and evolution.

[27] Wilkie AO (1994). The molecular basis of genetic dominance. Journal of medical genetics.

[28] Majer A, Blanchard AA, Medina S, Booth SA, Myal Y (2016). Claudin 1 Expression Levels Affect miRNA Dynamics in Human Basal-Like Breast Cancer Cells. DNA and cell biology.

[29] Matys V (2006). TRANSFAC and its module TRANSCompel: transcriptional gene regulation in eukaryotes. Nucleic acids research.

[30] Piva F, Giulietti M, Burini AB, Principato G (2012). SpliceAid 2: a database of human splicing factors expression data and RNA target motifs. Human mutation.

[31] Chakraborty, U. & Alani, E. Understanding how mismatch repair proteins participate in the repair/anti-recombination decision. *FEMS yeast research* **16** (2016). 10.1093/femsyr/fow071 PMC5976031 27573382

[32] Monteverde T, Muthalagu N, Port J, Murphy DJ (2015). Evidence of cancer-promoting roles for AMPK and related kinases. The FEBS journal.

[33] Sporn MB, Liby KT (2012). NRF2 and cancer: the good, the bad and the importance of context. Nature reviews. Cancer.

[34] Kanehisa M, Furumichi M, Tanabe M, Sato Y, Morishima K (2017). KEGG: new perspectives on genomes, pathways, diseases and drugs. Nucleic acids research.

[35] Kanehisa M, Sato Y, Kawashima M, Furumichi M, Tanabe M (2016). KEGG as a reference resource for gene and protein annotation. Nucleic acids research.

[36] Marra G (1998). Mismatch repair deficiency associated with overexpression of the MSH3 gene. Proceedings of the National Academy of Sciences of the United States of America.

[37] Kolinjivadi, A. M. *et al*. Moonlighting at replication forks: a new life for homologous recombination proteins BRCA1, BRCA2 and RAD51. *FEBS letters* (2017). 10.1002/1873-3468.12556 28079255

[38] Ciccia A (2014). Treacher Collins syndrome TCOF1 protein cooperates with NBS1 in the DNA damage response. Proceedings of the National Academy of Sciences of the United States of America.

[39] Saviozzi S (2009). Non-small cell lung cancer exhibits transcript overexpression of genes associated with homologous recombination and DNA replication pathways. Cancer research.

[40] Lee EYHP, Muller WJ (2010). Oncogenes and tumor suppressor genes. Cold Spring Harbor perspectives in biology.

[41] Tran, T. Q. *et al*. Tumor-associated mutant p53 promotes cancer cell survival upon glutamine deprivation through p21 induction. *Oncogene* (2016). 10.1038/onc.2016.360 PMC5383530 27721412

[42] Soussi T, Wiman KG (2015). TP53: an oncogene in disguise. Cell death and differentiation.

[43] Zhao L (2016). Multifunctional DDX3: dual roles in various cancer development and its related signaling pathways. American journal of cancer research.

[44] Wu GS (2007). Role of mitogen-activated protein kinase phosphatases (MKPs) in cancer. Cancer metastasis reviews.

[45] Lykke-Andersen S, Jensen TH (2015). Nonsense-mediated mRNA decay: an intricate machinery that shapes transcriptomes. Nature reviews. Molecular cell biology.

[46] Radhakrishnan A, Green R (2016). Connections Underlying Translation and mRNA Stability. Journal of molecular biology.

[47] Mayr C, Bartel DP (2009). Widespread shortening of 3′UTRs by alternative cleavage and polyadenylation activates oncogenes in cancer cells. Cell.

[48] Li, H., Wang, J., Mor, G. & Sklar, J. A neoplastic gene fusion mimics trans-splicing of RNAs in normal human cells. Science (New York, N.Y.) **321**, 1357–1361 (2008). 10.1126/science.1156725 18772439

[49] Lindeboom RGH, Supek F, Lehner B (2016). The rules and impact of nonsense-mediated mRNA decay in human cancers. Nature genetics.

[50] Karam R, Wengrod J, Gardner LB, Wilkinson MF (2013). Regulation of nonsense-mediated mRNA decay: implications for physiology and disease. Biochimica et biophysica acta.

[51] Gardner LB (2010). Nonsense-mediated RNA decay regulation by cellular stress: implications for tumorigenesis. Molecular cancer research: MCR.

[52] Frischmeyer PA, Dietz HC (1999). Nonsense-mediated mRNA decay in health and disease. Human molecular genetics.

[53] Lawrence MS (2013). Mutational heterogeneity in cancer and the search for new cancer-associated genes. Nature.

[54] Da Sylva TR, Gordon CS, Wu GE (2009). A genetic approach to quantifying human *in vivo* mutation frequency uncovers transcription level effects. Mutation Research - Fundamental and Molecular Mechanisms of Mutagenesis.

[55] Evans P, Avey S, Kong Y, Krauthammer M (2013). Adjusting for background mutation frequency biases improves the identification of cancer driver genes. IEEE transactions on nanobioscience.

[56] Watson IR, Takahashi K, Futreal PA, Chin L (2013). Emerging patterns of somatic mutations in cancer. Nature reviews. Genetics.

[57] Supek F, Lehner B (2015). Differential DNA mismatch repair underlies mutation rate variation across the human genome. Nature.

[58] Zhang J, Zhang S, Wang Y, Zhang X-S (2013). Identification of mutated core cancer modules by integrating somatic mutation, copy number variation, and gene expression data. BMC systems biology.

[59] Cho A (2016). MUFFINN: cancer gene discovery via network analysis of somatic mutation data. Genome Biology.

[60] Suo C (2015). Integration of somatic mutation, expression and functional data reveals potential driver genes predictive of breast cancer survival. Bioinformatics (Oxford, England).

[61] Edsgärd, D. *et al*. Geneiase: Detection of condition-dependent and static allele-specific expression from rna-seq data without haplotype information. *Scientific reports* **6** (2016). 10.1038/srep21134 PMC4758070 26887787

[62] Lu R (2015). Analyzing allele specific RNA expression using mixture models. BMC genomics.

[63] Narayan S, Bader GD, Reimand J (2016). Frequent mutations in acetylation and ubiquitination sites suggest novel driver mechanisms of cancer. Genome Medicine.

[64] Fischer K, Pflugfelder GO (2015). Putative Breast Cancer Driver Mutations in TBX3 Cause Impaired Transcriptional Repression. Frontiers in oncology.

[65] Zheng X (2014). MethylPurify: tumor purity deconvolution and differential methylation detection from single tumor DNA methylomes. Genome biology.

[66] Pages F (2010). Immune infiltration in human tumors: a prognostic factor that should not be ignored. Oncogene.

[67] Yoshihara K (2013). Inferring tumour purity and stromal and immune cell admixture from expression data. Nature communications.

[68] Aran D, Sirota M, Butte AJ (2015). Systematic pan-cancer analysis of tumour purity. Nature communications.

[69] Storchova Z, Kloosterman WP (2016). The genomic characteristics and cellular origin of chromothripsis. Current Opinion in Cell Biology.

[70] Stephens PJ (2011). Massive genomic rearrangement acquired in a single catastrophic event during cancer development. Cell.

[71] Trapnell C (2013). Differential analysis of gene regulation at transcript resolution with RNA-seq. Nature biotechnology.

[72] Thorvaldsdo´ttir H, Robinson JT, Mesirov JP (2013). Integrative Genomics Viewer (IGV): high-performance genomics data visualization and exploration. Briefings in bioinformatics.

[73] Krzywinski M (2009). Circos: an information aesthetic for comparative genomics. Genome Research.

[74] Fisher RA (1922). On the Interpretation of *χ*2 from Contingency Tables, and the Calculation of P. Journal of the Royal Statistical Society.

[75] Plackett RL (1983). Karl Pearson and the Chi-Squared Test. International Statistical Review/Revue Internationale de Statistique.

[76] Yates F (1934). Contingency Tables Involving Small Numbers and the *χ*2 Test. Supplement to the Journal of the Royal Statistical Society.

[77] Student. The Probable Error of a Mean. *Biometrika* **6**, 1–25 (1908).

