# Supplementary material for: Overexpressed somatic alleles are enriched in functional elements in Breast Cancer
Source: Sci Rep. 2017 Aug 15;7:8287. doi: 10.1038/s41598-017-08416-w (PMC5557904; doi:10.1038/s41598-017-08416-w)
Supplement: Supplementary file 1 — Supplementary Figures [file 41598_2017_8416_MOESM1_ESM.pdf]

Overexpressed somatic alleles are enriched in functional elements in Breast Cancer

Paula Restrepo,[1,2+] Mercedeh Movassagh,[2,3+] Nawaf Alomran,[2,4+] Christian Miller,[2,+] Muzi Li,[2,4+] Chris Trenkov,[2] Yulian Manchev,[2] Sonali Bahl,[1] Stephanie Warnken,[1] Liam Spurr,[1,2] Tatiyana Apanasovich,[5] Keith Crandall,[6] Nathan Edwards,[3] and Anelia Horvath[1,2,6,7*]

[1]Department of Pharmacology and Physiology, School of Medicine and Health Sciences, The George Washington University, Washington, DC 20037, USA

[2]McCormick Genomics and Proteomics Center, School of Medicine and Health Sciences, The George Washington University, Washington, DC 20037, USA

[3]University of Massachusetts Medical School, Program in Bioinformatics and Integrative Biology, Worcester, MA 01605, USA

[4]Department of Biochemistry and Molecular and Cellular Biology, Georgetown University, School of Medicine, Washington, DC 20057, USA

[5]Department of Statistics, The George Washington University, Washington, DC 20037, USA

[6]Department of Biochemistry and Molecular Medicine, School of Medicine and Health Sciences, The George Washington University, Washington, DC 20037, USA

[7]Computational Biology Institute, The George Washington University, Washington, DC 20037, USA

[*]Correspondence to: horvatha@gwu.edu

[+]these authors contributed equally to this work

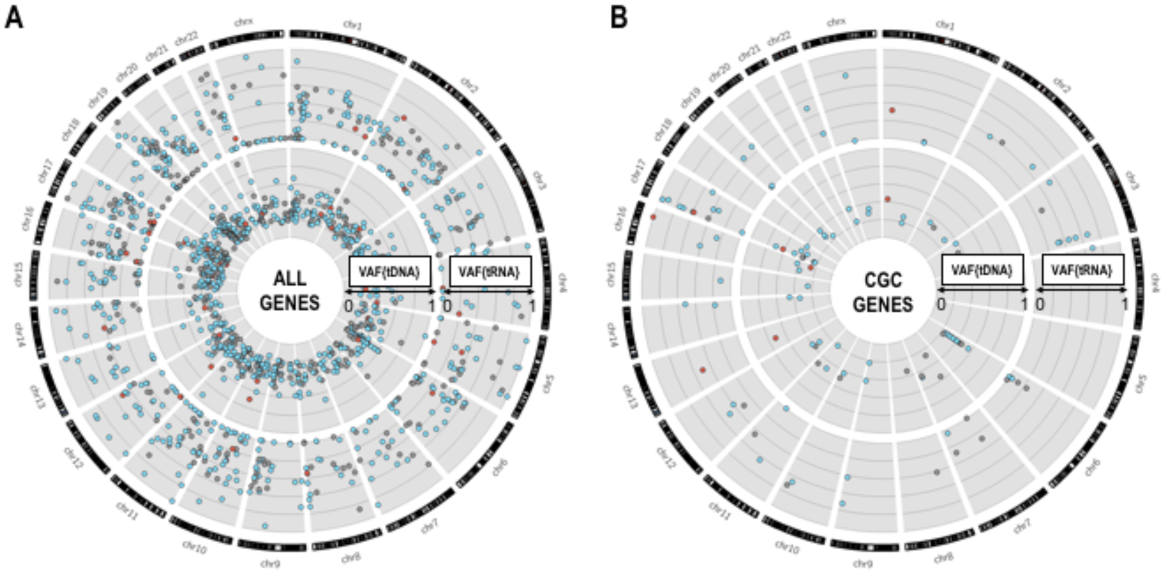

Supplementary Figure 1. Circos plots of VAF{tRNA} (outer layer) and VAF{tDNA} (inner layer) of somatic mutations called in the entire dataset (A) and in the CGC genes (B) in the 72 TCGA BRCA samples. VAF{tRNA} and VAF{tDNA} (scale 0 to 1, center-outwards). The different types of nucleotide substitutions are distinguished by colors: nonsense - red, missense - blue, and synonymous - gray.

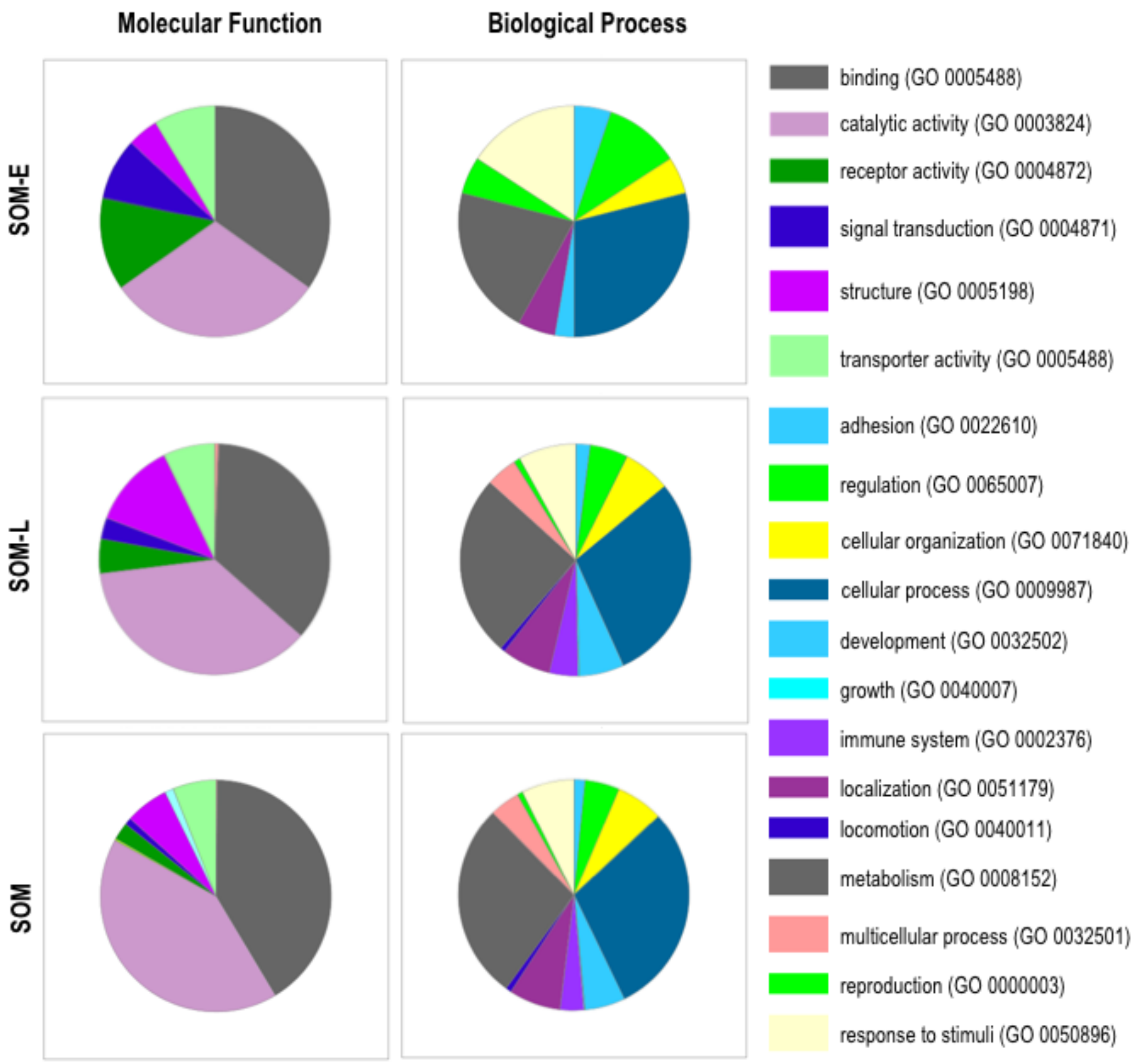

Supplementary Figure 2. Molecular function (left) and biological process (right) of genes hosting SOM-E, SOM-L and SOM variants (GO annotations).
